# School health and nutrition program implementation, impact, and challenges in schools of Nepal: stakeholders’ perceptions

**DOI:** 10.1186/s41182-019-0159-4

**Published:** 2019-05-14

**Authors:** Rachana Manandhar Shrestha, Mamata Ghimire, Prakash Shakya, Rakesh Ayer, Rolina Dhital, Masamine Jimba

**Affiliations:** 1 Department of Community and Global Health, Graduate School of Medicine, The University of Tokyo, 7-3-1, Hongo, Bunkyo-ku, Tokyo, 113-0033 Japan; 2 Department of Health Care Policy and Management, Graduate School of Comprehensive Human Sciences, University of Tsukuba, 1-1-1, Tennodai, Tsukuba, Ibaraki, 301-8577 Japan; 3 Graduate School of International Cooperation Studies, Kyorin University, 5-4-1, Shimorenjaku, Mitaka-shi, Tokyo, 181-8612 Japan

**Keywords:** School, School children, School health and nutrition program, Implementation, Impact, Challenges, Nepal

## Abstract

**Background:**

The School Health and Nutrition (SHN) program is a cost-effective intervention for resource-poor countries. SHN program aims to provide timely support and preventive measures to improve the health of school children, which can be associated with their cognitive development, learning, and academic performance. Stakeholders at different tiers can play significant roles in the program implementation and its success. Their perceptions are equally important to provide information on the factors influencing the implementation process and help to identify the gaps in the process. However, the evidence is scarce on the school health and nutrition policy and program implementation in developing countries. No study has yet explored stakeholders’ perceptions on the SHN program implementation process in low-income countries, including Nepal. Therefore, we conducted a qualitative study to explore (1) the SHN program implementation, (2) its impact, and (3) challenges in Nepal.

**Methods:**

We conducted a qualitative study through 32 in-depth interviews of the key informants who were actively involved in SHN program implementation in Nepal. The key informants were identified through personal network and snowballing procedure. We adopted a thematic approach for the data analysis.

**Results:**

We categorized interview data into three broad themes: (1) SHN program implementation, (2) its impact, and (3) challenges during implementation. Almost all the key informants appreciated the program for its positive impact on students, schools, and communities. The positive impacts included improved students’ health and school environment and enhanced community awareness. However, the key impediments in implementing the program included a lack of coordination between stakeholders, lack of resources, limited training opportunities, and doubts regarding the sustainability of the program.

**Conclusions:**

This study provided a deeper understanding of the linkage between the SHN program implementation, impact, and challenges in Nepal. Despite the challenges, all the stakeholders acknowledged that the SHN program had positive impacts on students, schools, and communities. Our findings highlighted that stakeholders from all tiers should coordinate, collaborate, and continue their efforts to effectively implement and expand the program nationwide. Awareness campaigns and advocacy for the program are indispensable to pull more resources from relevant stakeholders.

## Background

Schools have been a powerful setting to promote health programs [1, 2]. The School Health and Nutrition (SHN) program is a cost-effective intervention for resource-poor countries where more schools and teachers are available than health care institutions and workers [3, 4]. Many school-aged children in these countries are affected by treatable and preventable illnesses [3, 4]. School children’s ill health can be associated with poor cognitive development, learning, and academic performance [5, 6]. The SHN program aims to provide timely support and preventive measures to improve the health of school children [4, 7].

In 1995, the World Health Organization (WHO) launched the Global School Health Initiative and introduced the concept of Health-Promoting Schools (HPS) [8], which can be characterized as schools seeking to strengthen their capacity and enable a healthy environment in the schools [8]. The SHN program is an integral part of HPS [9], and many countries have adopted the SHN program to promote health through schools [10].

Health-promotion activities have been successfully implemented through the SHN program in resource-rich countries [1, 5, 11]. However, in resource-limited countries, several operational barriers exist to implement such programs. Major challenges identified include insufficient funds, inadequate physical infrastructures, and lack of trained human resources [3, 12–14]. Furthermore, poor coordination and partnerships between stakeholders are also significant hindrances [15].

In Nepal, the Ministry of Health (MOH) and the Ministry of Education (MOE) jointly endorsed the National SHN Strategy in 2006 [16]. Since the strategy was endorsed, the Government of Nepal has been conducting the SHN program in different parts of the country with technical and financial support from several aid agencies. The program aims to improve the physical, mental, emotional, and educational status of school children in Nepal [15, 16]. However, the coverage of the program activities has not reached many districts in the country [15, 17], and most of the support has been limited to the students of government schools [15, 17]. Besides, many stakeholders are conducting only selective activities [15].

To improve the quality of the SHN program, generating evidence is essential. With this aim, in 2013, we conducted an evaluation study of a 4-year-long SHN project jointly conducted by JICA and Nepal Government in Sindhupalchok and Syangja districts [18]. The project included SHN activities such as general and oral health check-ups, first aid services, deworming, iron supplementation, child club activities, and special health classes, maintaining the SHN register and providing mid-day meals. It also involved school cleaning; improving access to safe drinking water, toilet, and hand-washing facilities; and constructing toilets and waste disposal pits in schools. The teachers in the project schools were trained to conduct SHN activities. The results showed that students in project schools had better access to various school health services, hygiene and sanitation facilities, and more child club activities and special health classes compared to those in comparison schools. Eventually, better access to hygiene and sanitation facilities improved students’ hygiene practices, and their improved hygiene practices were associated with positive health outcomes [18].

The evidence is scarce on the SHN program implementation process in resource-limited countries [9, 12, 19], and no study has yet explored stakeholders’ perceptions on the SHN program implementation process in low-income countries, including Nepal. Therefore, while evaluating the SHN project, we also aimed to assess the SHN program implementation process in different parts of the country. In the SHN project evaluation study, we only collected data from the school students but did not include other stakeholders, who were actively involved in the SHN program implementation. However, stakeholders at different tiers can play significant roles in the program implementation and its success [20]. Their perceptions are equally important to provide information on the factors influencing the implementation process and help to identify the gaps in such processes [9, 21]. We thus conducted a qualitative study to explore (1) the SHN program implementation, (2) its impact, and (3) challenges in Nepal.

## Methods

### Study design and participants

In this qualitative study, we conducted 32 in-depth interviews with key informants from September to December 2013. We used a stratified purposive sampling technique to choose the study areas [22, 23] and included seven out of the then 75 districts. The seven districts were Siraha, Sindhupalchok, Syangja, Kailali, Kathmandu, Lalitpur and Bhaktapur. Kathmandu, Lalitpur, and Bhaktapur districts are in the Kathmandu valley, where MOE, MOH, and different aid agencies were located. Other four districts were selected because several aid agencies were implementing SHN programs in those districts [16, 17]. Furthermore, the seven districts represented three physiographic and four out of five the then developmental regions of the country.

### Study participants

Table 1 shows the number of key informant interviews conducted from different tiers of stakeholders. We included key informants who were actively involved in the SHN program development and implementation. We recruited the key informants through the personal network of a key person who was also actively involved in the SHN program development and implementation. Using his extensive network of connections and a snowballing procedure, we identified key informants from different organizations who had in-depth knowledge and were actively involved in the program. We conducted office visits and had telephone conversations, and formal and informal talks to track key informants.

**Table 1:** Districts, key informants, and number of interviews

SN	Key informants	Districts	No. of interviews
1.	Central level (focal person for SHN program)	Kathmandu, Lalitpur, and Bhaktapur	
	Ministry of Health		3
	Ministry of Education		2
2.	Aid agency (focal person for SHN program)	Kathmandu and Lalitpur	7
3.	District level (focal person for SHN program)	Sindhupalchok, Syangja, Siraha, and Kailali	
	District education office		4
	District health office		4
4.	School	Sindhupalchok, Syangja, Siraha, and Kailali	
	Focal teacher/school principal		8
	Local NGO/resource person/SMC member		4

The key informants represented four different levels: (1) central level and (2) aid agencies in Kathmandu valley and (3) district level and (4) schools in Siraha, Sindhupalchok, Syangja, and Kailali districts. At the central level, we included two key informants from the Department of Education, MOE, and three from the Child Health Division, MOH. The central-level key informants were involved in the SHN program development, networking, resource mobilization, and monitoring. From the aid agencies, we recruited one key informant from each of seven different international non-governmental organizations (INGOs), and UN and bilateral organizations. The key informants from the aid agencies were involved in supporting the MOE and MOH to implement the program as well as in monitoring and supervising the programs implemented in the schools. At the district level, four key informants from four District Health Office and four District Education Office were recruited from Siraha, Sindhupalchok, Syangja, and Kailali districts. The district level key informants were involved in planning, coordination, resource mobilization, and monitoring of the SHN program in the schools. From the schools also, we recruited four school principals, four teachers, two local non-governmental organizations (NGO) members, one resource person, and one school management committee member from Siraha, Sindhupalchok, Syangja, and Kailali districts. Key informants from the schools implemented and self-monitored the SHN program in the schools.

### Data collection and interview guide

The first author and a research assistant conducted all the interviews at the key informants’ workplace, some in English and some in Nepali language. Each interview lasted for an average of 1 h and was tape-recorded and transcribed. Notes were also taken while interviewing. At the end of each interview, the interview notes were reviewed with each key informant to validate what he or she intended to convey. After we felt that data saturation was reached, we stopped the data collection procedure [24]. We followed the consolidated criteria for reporting qualitative research (COREQ) guidelines to conduct the interviews, and analyze and report the data [25].

We used a modified interview guide based on the Policy Implementation Assessment Tool for program implementers and other stakeholders, developed by the United States Agency for International Development (USAID) [26]. The interview guide included open-ended questions and has been used in health policy and program analysis in several low- and middle-income countries [27]. The guide was translated into Nepali and back translated into English by different individuals to ensure the quality of the translated version.

### Data analysis

We conducted a thematic analysis, an inductive approach, using the conceptual framework developed by USAID [28] to analyze the data from the in-depth interviews. The framework has been designed to show the links between health-related policy development, program implementation, and health outcomes. We employed this framework to identify themes, codes, and sub-codes from the data and analyzed them to understand the data patterns [29]. We then analyzed the data following the 5-phase cycle proposed by Yin [23], which includes (1) compiling, (2) disassembling, (3) reassembling, (4) interpreting, and (5) concluding.

In the compiling phase, the research assistants transcribed the interviews. The first author (RMS) then assigned unique code numbers to all the transcripts from the 32 key informants as P1 to P32 (P refers to participant), verified the transcripts with the tape-recorded conversations and written notes.

In the disassembling phase, the first author thoroughly read and reread the transcripts, listened to all the interviews repeatedly, and examined the patterns of interview data. The first author then identified the specific data segments, which were related to study objectives, and gave labels to the data segments to develop preliminary themes, codes, and sub-codes based on the conceptual framework by USAID [28]. The group of codes and sub-codes, which repeated in a patterned way, were grouped into a theme. The first author then discussed themes, codes, and sub-codes with co-authors. We imported the translated texts into Atlas.ti software, version 5, for data sorting and coding. We distributed the 32 transcripts equally between two groups of co-authors. In each group, two co-authors separately read, sorted, and coded 16 transcripts into previously formulated themes, codes, and sub-codes and then tallied their results to reach consensus. The first author also sorted and coded all 32 transcripts separately. Subsequently, each group tallied their results with the first author’s results to deduce the final themes, codes, and sub-codes.

In the reassembling phase, we reassembled all the data under the same themes, codes, and sub-codes into different groups. In the interpreting phase, we wrote summaries to interpret the data and discussed important quotations. Three co-authors summarized each transcript, selected quotations, and translated them into English. Finally, in the concluding phase, the first author read the summaries and selected quotations to draw conclusions from the data and discussed them with all co-authors.

### Ethical considerations

The Research Ethics Committee of the University of Tokyo and the Nepal Health Research Council (NHRC) approved this study. We obtained written informed consent from all the key informants before the interview. We informed them that their participation was voluntary and they could withdraw from the study at any time. We also assured them of confidentiality and anonymity.

## Results

We categorized the interview data into three broad themes: (1) SHN program implementation, (2) impact of the SHN program, and (3) challenges during program implementation and suggestions from stakeholders [28]. Table 2 shows the major themes, codes, and sub-codes deduced from the thematic analysis, showing the linkage between program implementation, impact, and challenges during implementation.

**Table 2:** Themes, codes, and sub-codes used for data analysis

Themes	Codes	Sub-codes
1. SHN program implementation	a. Stakeholders involved in SHN program implementation	
	b. Major SHN activities	1. Improve use of SHN services 2. Improve school environment 3. Improve health and nutritional knowledge and behaviors 4. Improve in community support system and policy environment
2. Impact of the SHN program	a. Impact on student b. Impact on school environment and community	
3. Challenges in program implementation and suggestions from stakeholders	a. Lack of coordination between stakeholders b. Limited financial, human and material resources c. Limited training opportunities d. Sustainability of the program e. Suggestions from the stakeholders	

### SHN program implementation

#### Stakeholders involved in SHN program implementation

Majority of the participants from the central, aid agencies, and district level mentioned that there is a structural network from top to down, which included Department of Health Services (MOH), Department of Education (MOE), and different aid agencies at the national level, which were involved in program implementation. At the district level and schools, depending upon the local context and area, the key players involved were District Health Office, District Education Office, schools, Village Development Committee, District Development Committee, local NGOs, health posts, Female Community Health Volunteers (FCHVs), youth clubs, and parents. A few participants from the central level stated about the SHN network, which was also formed with stakeholders from different tiers and has been actively involved in implementing SHN programs as a campaign.

At the central level, the ministry of education, ministry of health, national planning commission and different aid agencies are involved, while at grass root level district health office and education office, schools, school management committee, child clubs, parents, students unions, health posts etc. are the main stakeholders actively involved." (P3, aid agency: WASH specialist)

Now, the SHN network is formed and all the stakeholders involved in it have planned and divided their responsibilities. None of the organizations go directly for the implementation of SHN. We all go through SHN network, which has been a good mechanism where we can coordinate." (P1, aid agency: SHN program coordinator)

#### Major SHN activities

According to the key informants’ responses from all levels, the major SHN activities could be mainly categorized into four sections, which were based on the four objectives of the SHN strategy [16]. The activities aimed at achieving these objectives are listed below.

##### Improve use of SHN services by school students

Majority of key informants responded that they conducted activities such as general and oral health check-ups, first aid services, deworming, iron supplementation, child club activities, maintaining the SHN register, and providing mid-day meals. These activities aimed to improve students’ use of SHN services.

We particularly focused on health examination, oral health check-ups and camps, tooth brushing and hand washing every day, providing mid-day meals, first aid training, providing first aid box to schools, providing training to school teachers and child clubs in the schools." (P1, aid agency: SHN program coordinator)

##### Improve school environment

Majority of key informants from all levels mentioned that they conducted activities such as school cleaning programs, access to safe drinking water, improving toilet and hand washing facilities, waste disposal pits in school, construction of classrooms, toilets, etc. They mentioned that the above activities helped to improve the school environment.

"Students used to defecate openly in the past, but now they have started using toilets. They collect garbage in the garbage box and after it is filled, they burn it." (P28, school: Health and physical education teacher)

##### Improve health and nutritional knowledge and behaviors of students

According to the key informants from aid agencies, district level, and schools, they conducted activities such as health education classes, child clubs, and extra-curricular activities to improve students’ health-related knowledge and behaviors. Besides, schoolteachers and child club members were trained to conduct health education sessions on SHN. Awareness programs for parents and community were also conducted.

After the SHN program started, there have been many improvements. For example, this program has improved students’ knowledge of health and hygiene practices and keeping the school environment clean, etc. We have seen many positive changes after this program. (P21, school: Resource person for SHN program)

##### Improve community support system and policy environment

Some key informants from the central level and aid agencies stated that at the central level, the members of the SHN network and government actively participated in regular meetings to share and discuss the SHN program strategies, activities, and achievements. Some key informants also mentioned that they received support from communities to conduct SHN activities effectively.

At the central level, we are the active members of the SHN network. So we are actively participating in the program. (P3, aid agency: WASH specialist)

Water facility was not available in the schools in Pyuthan district. We had a meeting with parents and teachers and told the parents that we could just give them pipelines. Then, they did all the labor work to set up the pipelines. This is one good example of cooperation between school and community. (P4, aid agency: SHNP senior coordinator)

### Impact of the SHN program

Based on the key informants’ responses, we categorized the impact of the SHN program into two main parts a) impact on students, and b) impact on school environment and community.

#### Impact on students

All the key informants in this study mentioned that the SHN program was successful in improving students’ general knowledge of health and nutrition. Furthermore, the program also brought positive changes in students’ nutritional behaviors, hygiene practices, and life skills. Some participants also appreciated providing tiffin boxes to students, after which many parents started sending tiffin from home in those tiffin boxes.

Students’ awareness on hygiene and sanitary practices has improved. When I was in Dadheldhura, I visited one of the schools there. When I was looking for a toilet, one of the students from grade 3 showed me the toilet and hand washing soap. (P15, district level: SHN program officer, District Education Office)

The tiffin box program would be one of the success stories and good practices. Parents started sending tiffin to their kids in these tiffin boxes. So this is about the behavior change among students as well as their parents. (P5, aid agency: Country program coordinator, School Feeding Program)

Many key informants further stated that the program improved students’ health status by reducing problems such as diarrhea, parasitic infections, anemia, blindness, and hearing loss.

When we conducted the program, we had the baseline and end-line data, which showed a huge reduction in anemia. (P7, aid agency: SHNP former chief advisor)

In the past, there used to be diarrhea epidemics but now there are no such incidents. (P18, district level: District Public Health officer, District Health Office)

Physical screening has helped us to identify vision and hearing impairments. We have prevented kids from becoming blind after vision screening and referring them for further treatments. We have also prevented some kids from suffering permanent hearing loss. (P4, aid agency: SHNP senior coordinator)

Many key informants also mentioned that the program improved the attendance, enrolment, and retention rates in schools. They further suggested that improved health has a positive impact on students’ academic performance.

In the past, students could not understand what they were taught. It’s because their stomach used to be empty. So their focus was more on their empty stomach than on their study. But now all the students bring tiffin. Even if they forget to bring tiffin, their parents bring it to school. So students do not run away from their schools. Their health condition is also getting better. (P29, school: SHN program coordinator, local NGO)

We have qualitative data and reports, which showed students now want to come to school and don’t go back in a break. We have mid-day meal promotion so students come back. For adolescents, we have menstrual hygiene management class, which brings them to school. (P4, aid agency: SHN program senior coordinator)

#### Impact on school environment and community

Majority of key informants mentioned that the SHN program brought positive changes in the school environment and community. The cooperation between schools and the community also improved. In some areas, communities were mobilized in SHN activities, leading to community awareness.

Children are changing agents. They are promoting health and hygiene not only in their schools but also in their homes and communities. (P3, aid agency: WASH specialist)

In the past, proper coordination and communication did not exist between schools and communities, so the communities used to be dirty with open defecation. Even tooth brushing was neglected. After the child club mobilization, the child club members conducted rallies in the villages to generate awareness among the community people. After receiving the messages from the school children, the communities have been empowered. The open defecation decreased and more toilets were built. Later, an open-defecation-free zone was declared in school catchment areas. Parents have also started brushing their teeth! (P1, aid agency: SHNP program coordinator)

### Challenges in program implementation and suggestions from the stakeholders

#### Lack of coordination between stakeholders

Majority of key informants responded that MOH, MOE, and their institutions from the central to local levels were responsible to implement the programs and a certain level of coordination existed between them. However, some of the key informants from the central level and aid agencies mentioned that MOH was more active compared to the MOE. Furthermore, the overall coordination between these two sectors was limited, which therefore led to a lack of planning for the sustainability and scaling up of the program.

There are some difficulties with coordination among stakeholders. Horizontal coordination is more difficult than vertical coordination. (P7, aid agency: SHNP former chief advisor)

Most of the organization and ongoing activities come from the health sector. Lower numbers of NGOs or INGOs working in the education sector are involved in SHN program implementation. (P4, aid agency: SHNP senior coordinator)

Though a certain level of coordination exists between the stakeholders, I don’t see that extent of coordination even at the central level, which could generate resources. So I think it is a bit lacking in this part, which can be a challenge for the sustainability of the program (P5, aid agency: SHNP head)

#### Limited financial, human, and material resources

Almost all key informants in this study responded that the allocated funds for the SHN program were not sufficient to implement all the program components and expand it nationwide. Besides, insufficient human resources and physical infrastructures were other major hurdles. Many key informants from the aid agencies agreed that they have limited resources and could conduct only selected programs in some target districts.

We conducted the SHN project from Japan International Cooperation Agency's (JICA) support. But we are facing difficulties to expand the program because of financial problems. (P9, central level: Director, Child Health Division)

Human and material resources are insufficient from the central to the district level. We have not been able to fulfill the demands. (P8, central level: Chief, Nutrition Section at Child Health Division)

In our school, we do not have teachers with enough knowledge about health issues. Also, we have not been able to use toilets properly and they are smelly because of lack of water facilities. (P26, school: Chairperson, School Management Committee)

By using available funds, we can conduct activities that only meet the indicators proposed by our headquarters. We do not and cannot do all the activities of the SHN program. (P1, aid agency: SHN program coordinator)

#### Limited training opportunities

All the key informants agreed that training is essential to implement the SHN program effectively. Though the majority of the key informants from the central level and aid agencies stated that they have received different trainings, mixed responses were obtained from the key informants at the district level and schools. Some of them mentioned that they had received the training once, while some were not even aware of such training.

The training was conducted only once and ended and it was not repeated. (P23, school: Health and physical education teacher)

I am the focal person for the SHN program. I have worked in the health training department for 7 years but haven’t received any training related to school health yet and I also don’t know about it. (P16, district level: Chief district officer, District Health Office)

Some key informants from the central and aid agencies responded that the lack of trained human resources and turnover of trained staff members were also impediments during program implementation.

As soon as he/she gets some training, he/she will be transferred somewhere else due to either personal interest or organizational changes. (P12, central level: Deputy Director of Education Division)

#### Sustainability of the program

Almost half of the key informants from different levels were positive regarding the sustainability of the program, while others were doubtful due to lack of resources.

It is not sustainable. We don’t have enough resources. We have conducted it in two districts but could not expand it to other districts. So if resources are available, we can make it sustainable. (P9, central level: Director, Child Health Division)

#### Suggestions from the stakeholders

Despite several challenges identified by the stakeholders during the implementation of SHN programs, all of them acknowledged that efforts should be made to make the program sustainable, because of its positive impact on students, schools, and communities. Some of the key informants from the central level and aid agency suggested that MOE should also get actively involved in the program implementation. Besides, a few respondents at the central level mentioned that the program could be sustainable if it is integrated into the government system.

The education sector should be more involved, as the Ministry of Education is also on board. (P4, aid agency: SHNP senior coordinator)

The program will be sustainable if it is integrated into the government system. Aid agencies come and once they are gone, the District Education Office and communities cannot make the program sustainable by themselves. (P4, aid agency: SHNP senior coordinator)

Some of the key informants also provided suggestions on resource generations. One of the key informants from the aid agency mentioned that the stakeholders should coordinate well to generate resources. Some key informants from the schools even mentioned that they tried to generate funds from local sources.

In my opinion, the coordination between the stakeholders should also act on pulling up the resources for implementing the program. (P5, aid agency: SHNP head)

Child clubs in schools organize Deusi-Bhailo program (cultural program) during Tihar festival and collect fund. The child clubs also charge membership fees to generate fund, which they use for school health and nutrition program activities (P23, school: Resource person for SHN program)

Regarding training on SHN program implementation, stakeholders at districts and schools who had received training on SHN program implementation once, suggested that such training should be more frequent and longer and also mentioned that it should be expanded to other parts of the country. They also suggested that all the teachers in the school should be trained.

The training was conducted only once and such training should be conducted repeatedly. (P23, school: Health and physical education teacher)

We need basic training for all teachers. I don’t think training only one focal teacher is sufficient. (P27, school: School principal)

## Discussion

In this study, almost all the key informants appreciated the SHN program implementation in schools and the positive impact it has on students, schools, and communities. The positive impact included improved students’ health and education outcomes, improved school environment, and enhanced community awareness. However, key informants also identified key impediments in implementing the program: there was a lack of coordination between stakeholders, lack of resources, limited training opportunities, and doubts regarding the sustainability of the program.

### SHN program implementation and impact

According to many key informants in this study, a broad array of stakeholders was involved from the central level to schools in implementing the SHN programs based on the SHN strategy in the country. MOH and MOE were the lead institutions for implementing the program. Aid agencies were also playing significant roles in implementing programs in different parts of the country. At district level and schools, DOH, DOE, schools, health posts, local NGOs, FCHVs, youth clubs, and parents were actively involved. Understanding the roles of different stakeholders is essential to analyze the implementation process of a program [30].

The majority of key informants mentioned that after the implementation of SHN program in the schools, students had better access to different SHN services, better nutrition, safe drinking water, and hygiene and sanitation facilities. They also acknowledged that the program significantly improved students’ knowledge, awareness, and practices regarding health and hygiene issues. The improved practices included hand washing, using soap while hand washing, and wearing clean school uniforms. Child clubs and extra-curricular activities could have played a significant role in improving students’ health and nutritional knowledge and practices. Similar child club activities are known to help students gain knowledge and learn life skills for their personal development in Nepal [31]. Furthermore, better access to hygiene and sanitation facilities in schools due to the SHN program could be associated with students’ better hygiene practices. Our previous quantitative study also showed that child clubs and special health classes were positively associated with students’ higher health knowledge scores and identified a positive association between better health and sanitation facilities and students’ improved hygiene practices [18]. The SHN program has also shown short- and long-term positive impact on students’ attitude, practices, health, and academic outcomes worldwide [10, 32, 33].

Many key informants also mentioned that the program had a positive impact on students’ health status, such as reduced diarrheal diseases, worm infestations, and anemia. Students’ better access to SHN services such as deworming, and iron and vitamin A supplementation might have played a significant role in the improved health outcomes. According to a few key informants, physical screening could have also prevented blindness and hearing loss in some students. Our previous study also showed a positive association between the SHN program and students’ better health outcomes [18]. Moreover, many key informants reported that students’ school enrolment, retention, and attendance rates increased after implementation of the SHN program. This finding may imply that healthy students attend school more regularly and stay longer in schools, which can have a positive impact on their academic performance [34, 35].

Furthermore, this study showed that after implementing the SHN program in schools, more parents sent their kids to school with tiffin and wearing a clean uniform. School children might have played a role as changing agents and generated awareness about nutrition, personal hygiene, and cleanliness at home and in their communities. These findings indicate that SHN program has helped to sensitize parents and community members about child health-related issues and promoting healthy behaviors; therefore, they also benefited from the program. Moreover, parents and community members can also play a significant role in encouraging children to practice healthy behaviors and keep their school environment clean, safe, and healthy [36]. Similar findings were also reported in the end-line survey of the SHN project conducted in Sindhupalchok and Syangja districts [37].

### Challenges in program implementation and suggestions from the stakeholders

Despite the positive impact of the SHN program on students, parents, and communities, this study identified several barriers and challenges to implement the program. Some of the key informants from the central and aid agency mentioned that horizontal coordination was lacking between the MOH and MOE. According to the institutional framework of the SHN strategy, the two ministries were the lead institutions to implement the program in Nepal, and aid agencies were the key implementing partners [16]. However, some key informants mentioned that the MOH and its institutions at the central to local level were more active compared to the MOE. This suggests that only one sector was actively involved in the implementation of the SHN program in Nepal. A similar situation was reported in the Lao People’s Democratic Republic (PDR), where the education sector had a leading role in implementing the National School Health Policy in the country [12]. A few studies have also reported a lack of coordination between the two ministries while implementing school health programs [9, 12, 13]. This gap could have led to the lack of intensive planning at national level which might be one of the reasons why the program could not be scaled up in other parts of the country as expected [12]. However, a few key informants in this study mentioned that regular meetings were held among the SHN network members at the central level to discuss program activities, achievements, and problems. These meetings might be helpful to improve program implementation [38].

Furthermore, most of the key informants identified insufficient funds and lack of material resources as the major hurdles to implement a comprehensive and nationwide SHN program. Many schools did not have sufficient physical infrastructures or facilities to implement the program efficiently. In resource-limited countries, a lack of resources has been a crucial operational barrier to conduct the program [3, 12, 13]. Our findings suggest that the SHN program in Nepal was not an exception. Aid agencies were one of the main sources of funding for the program in the country. However, the key informants from aid agencies also mentioned that they only had funds to implement the program in their target districts. They suggested that good coordination between the stakeholders might help in generating resources. Because of insufficient money, some schools even raised funds from parents and community members. This finding is encouraging and suggests that mobilizing community members to generate resources at the local level and reduce over-dependence on external aid agencies [14] may be effective to sustain SHN activities in Nepalese schools.

This study further showed that the lack of trained human resources to implement SHN was another key impediment. Although most of the key informants from the central level and aid agencies received and provided training to implement the SHN program, only some key informants from the district level and schools received the training. A few of them were not even aware of such training programs on SHN activities. Besides, only one focal teacher in each school was trained to conduct SHN activities and the students did not have access to the trained health professionals at schools. These findings indicate the dearth of trained human resources to conduct the program effectively. However, capacity building of human resources from the central level to schools is known to be a requisite to improve and sustain the program [12, 39]. A review study also reported that school health promoters required more training to overcome problems while implementing the health-promoting school programs [38]. During the 4-year SHN project in Sindhupalchok and Syangja districts, teachers and staff from the District Education Office and District Health Office were trained once to conduct SHN program activities [37]. In the present study, many key informants responded that after the project ended, such training was not conducted anymore. Moreover, a few of them identified the turnover of trained staff as a challenge, which is similar to the findings of a study from the Lao PDR [12]. Therefore, our findings warrant the need for regular and refresher training, and the establishment of training centers to generate trained manpower to implement the SHN program effectively and sustainably.

This study showed that the sustainability of the SHN program was a challenge because of insufficient material and human resources, and lack of strong leadership. A few of the key informants suggested integrating the program into the government system to make it sustainable. Program sustainability depends on the government’s strong leadership, long-term funds, and trained human resources [40, 41]. Despite the challenges, all the key informants in this study unanimously agreed to make efforts to increase the program sustainability, given its positive impact on students, schools, and communities. Some key informants even mentioned that in some schools, communities provided their support and took the initiative to conduct SHN activities, which suggests that communities can play a significant role in making the program sustainable.

### Limitations

The results of this study should be interpreted considering three limitations. First, we collected data from school-level key informants only in the districts where the SHN program was conducted. Therefore, key informants’ responses in this study may not represent stakeholders’ perceptions throughout the country. However, we interviewed key informants from different tiers including the central and district levels, schools, and aid agencies. Second, we did not include parents as key informants in this study though they are important stakeholders in SHN program implementation and their perceptions might have given additional insights. However, they were the least active stakeholders in implementing the program, and thus we have tried to understand their perceptions from school principals and focal teachers, who were communicating with parents on a regular basis. Third, the key informants might have expressed the views that they thought we (investigators) wanted to hear, leading to social desirability bias. However, we conducted interviews in the closed room in their office settings. We also assured them about the confidentiality of the information they provided and the anonymity of their identity. Despite these limitations, this is the first study in Nepal to explore the perceptions of stakeholders from all tiers regarding the SHN program implementation, impact, and challenges.

## Conclusions

This study provided a deeper understanding of the linkage between the SHN program implementation, impact, and challenges in Nepal. Stakeholders from all tiers identified several operational barriers to implementing and expanding the program throughout the country. The four major challenges identified by the stakeholders were lack of coordination between stakeholders, lack of resources, lack of training opportunities, and low sustainability of the program. Despite these challenges, all the stakeholders acknowledged that the SHN program had positive effects on students, schools, and communities and provided some suggestions to improve the implementation of SHN program in the country.

Our study further highlighted that stakeholders from all tiers should coordinate and collaborate adequately to continue their efforts to implement and expand the program nationwide. Furthermore, MOH and MOE should jointly provide strong leadership and recognize their responsibilities to improve students’ health and academic outcomes. Awareness campaigns and advocacy for the program are indispensable to pull more resources from relevant stakeholders. Besides, the government should implement programs to encourage schools to generate resources at the local level and discourage over-dependency on external sources to make the SHN program sustainable.

## Abbreviations

COREQ: Consolidated criteria for reporting qualitative research
FCHVs: Female Community Health Volunteers
HPS: Health-Promoting Schools
INGOs: International non-governmental organizations
MOE: Ministry of Education
MOH: Ministry of Health
NGOs: Non-governmental organizations
SHN: School Health and Nutrition
USAID: United States Agency for International Development
WHO: World Health Organization
